# Decoding FDA Labeling of Prescription Digital Therapeutics: A Cross-Sectional Regulatory Study

**DOI:** 10.7759/cureus.84468

**Published:** 2025-05-20

**Authors:** Shaheen E Lakhan

**Affiliations:** 1 Medical Department, Click Therapeutics, Inc., New York, USA; 2 Department of Neurology, Western University of Health Sciences, Pomona, USA; 3 Department of Neurology, A.T. Still University School of Osteopathic Medicine in Arizona, Mesa, USA; 4 Department of Neurology, Morehouse School of Medicine, Atlanta, USA; 5 Department of Bioscience, Global Neuroscience Initiative Foundation, Miami, USA

**Keywords:** 510(k) pathway, cognitive behavioral therapy, de novo classification, digital health regulation, fda clearance, icd-11 mapping, prescription digital therapeutics, regulatory labeling, software as a medical device, therapeutic indications

## Abstract

Background

Prescription digital therapeutics (PDTs) are software-only, FDA-regulated medical devices prescribed to prevent, manage, or treat disease. Despite increasing FDA clearance, there remains limited understanding of how PDTs are regulated and labeled from a product, sponsor, and indication standpoint.

Objective

This study aims to conduct the first systematic regulatory labeling analysis of all FDA-cleared PDTs, characterizing their approval pathways, sponsor profiles, clinical indications, and therapeutic language.

Methods

We performed a retrospective descriptive analysis of all software-only PDTs cleared by the FDA as of May 2025. Publicly available decision summaries, classification orders, and device listings were reviewed. Each PDT was examined by regulatory pathway, reviewing office, product code, sponsor geography, ICD-11 mapping, and FDA-approved labeling language, with a focus on terms of therapeutic intent and age-based eligibility.

Results

Thirteen PDTs were identified, with eight (61.5%) cleared via the 510(k) pathway and five (38.5%) via de novo classification. The most targeted neurological or psychiatric conditions were reviewed by the corresponding FDA offices. Sponsors were all US-based and concentrated in digital health hubs, particularly San Francisco. Therapeutic indications ranged from insomnia and diabetes to migraine and opioid use disorder. Labeling language varied: 11 PDTs included treatment claims, although most used modifiers such as “symptom improvement” or "aid in the management." Only one PDT, CT-132 for migraine, received a clean treatment label, defined as unambiguous treatment language without qualifiers, reflecting direct disease-targeting intent. Two PDTs deviated notably: reSET-O was labeled solely to increase outpatient treatment retention for opioid use disorder, and EndeavorRx was indicated to improve attention function without claiming to treat ADHD. Age-based eligibility spanned pediatric to adult definitions, consistent with FDA device criteria.

Conclusions

This study reveals meaningful variation in how PDTs are classified, labeled, and geographically distributed. FDA-sanctioned language plays a critical role in defining therapeutic scope and impacts how PDTs are interpreted by clinicians, payers, and patients. These insights mark a step forward in understanding the regulatory architecture of digital medicine.

## Introduction

Prescription digital therapeutics (PDTs) are software-based medical devices that deliver evidence-based interventions to prevent, manage, or treat disease [1]. Unlike general wellness apps or digital health tools, PDTs require a prescription and are regulated by the U.S. Food and Drug Administration (FDA), often following rigorous clinical validation. The requirement of a prescription signifies that PDTs operate within formal medical care pathways, involving clinician supervision, diagnosis-based eligibility, and integration with broader treatment plans, setting them apart from non-prescription wellness tools. As a novel therapeutic modality, PDTs have gained regulatory traction across a range of indications, including mental health, neurological disorders, metabolic disease, and functional syndromes.

The FDA has classified PDTs under the broader regulatory umbrella of software as a medical device (SaMD), primarily overseen by the Center for Devices and Radiological Health (CDRH) [2]. Unlike drugs, SaMDs are regulated as devices under a distinct framework that often requires different forms of evidence, shorter development timelines, and device-specific review criteria, all of which have implications for clinical trial design, labeling strategy, and reimbursement pathways. PDTs may enter the market via the 510(k) pathway using predicate devices or through de novo classification for first-of-a-kind technologies. However, unlike pharmaceuticals, PDTs operate within evolving regulatory and clinical frameworks, with variation in how their intended use, patient population, and therapeutic claims are articulated on FDA labeling [3].

While several reviews have discussed the clinical promise of digital therapeutics [4], there remains a lack of systematic analysis of FDA-cleared PDTs from a regulatory science perspective. Specifically, few studies have examined how PDTs are categorized by FDA product codes, how predicate device lineage influences market entry, or how labeling language shapes therapeutic positioning [5]. Understanding these regulatory patterns is essential for developers, clinicians, and policymakers as the digital therapeutics market matures. In particular, there remains limited analysis of the labeling language used in FDA-cleared PDTs, despite its significance in shaping clinician perception, payer coverage decisions, and patient engagement.

This study aims to provide a comprehensive analysis of all FDA-cleared PDTs to date, characterizing their regulatory pathways, sponsor characteristics, therapeutic indications, and FDA-approved labeling. Through structured analysis and classification of public FDA documentation, this work offers new insights into the regulatory architecture that defines and differentiates PDTs in the U.S. healthcare system.

## Materials and methods

Data sources and collection

This study employed a retrospective, descriptive analysis of all PDTs that received clearance from the FDA as of May 2025. Data collection was conducted between May 1 and May 7, 2025, using publicly available FDA resources, including the de novo classification requests, 510(k) premarket notifications, and medical device classification listings. Relevant decision summaries, classification orders, and FDA-issued labeling were reviewed in full to extract regulatory, administrative, and device-level information [6-18]. To ensure consistency and accuracy, official records were cross-referenced using the FDA’s Device Classification and Clearance portals [19]. Because the analysis focused on clearances issued prior to May 2025 and used cumulative FDA records, the short data collection window did not impact completeness.

Inclusion and exclusion criteria

The inclusion criterion was limited to PDTs, defined as software-only medical devices that had received FDA clearance with an explicitly defined prescription use. Excluded were non-prescription digital health products, general wellness tools, and software functions that support but do not deliver therapeutic intervention. Also excluded were software as a medical device (SaMD) that required hardware external to a smartphone to function, such as virtual reality headsets (i.e., Luminopia One; Luminopia Inc., Cambridge, MA, USA) or wearable physiological monitors (i.e., NightWare; Nightware, Inc., Hopkins, MN, USA) [20-21]. These devices were excluded because the inclusion of hardware components introduces additional regulatory considerations, such as performance testing and hardware risk assessments, that fall outside the scope of software-only PDT analysis.

Data extraction and processing

Each included PDT was cataloged across a structured set of variables encompassing regulatory pathway, grant and application dates, reviewing FDA division and office, product code, regulation name, sponsor identity and location, and any predicate device cited in the regulatory clearance. Initial data extraction and processing were performed using Python (Python Software Foundation, Wilmington, DE, USA), followed by manual review and verification. Microsoft Excel (Microsoft Corporation, Redmond, WA, USA) was used for data cleaning, review, and tabular organization. Manual review and verification were conducted by the author. As this was a single-reviewer study, inter-reviewer reliability was not assessed.

Sponsor names and device identifiers were normalized to remove punctuation and capitalization inconsistencies. In addition, device names were updated when the FDA-listed name at the time of regulatory filing differed from the subsequently marketed name (e.g., BT-001 updated to AspyreRx; Better Therapeutics Inc., Monroeville, PA, USA).

Condition mapping

To facilitate clinical categorization, the labeled indication for use provided by the FDA was extracted verbatim and analyzed to derive the primary therapeutic condition targeted by each PDT. These conditions were then systematically mapped to corresponding International Classification of Diseases 11th Revision (ICD-11) codes using the World Health Organization’s ICD-11 browser [22]. In cases where labeling language included multiple potential uses or vague symptom descriptions, we prioritized the primary indication as stated in the FDA’s official "Indications for Use" section for mapping to ICD-11 codes.

Analytical domains

The analysis was structured to explore patterns across seven major domains: regulatory trajectory and timing of market entry; FDA regulatory language and predicate use; division-level assignment of FDA review; sponsor-level characteristics and geographic distribution; therapeutic area categorization by labeled indication; ICD-11 classification alignment; and textual analysis of regulatory language used in indication statements. Special attention was paid to terms reflecting therapeutic intent (e.g., “treat,” “manage,” “reduce symptoms,” “aid in”) and to any population-specific restrictions, including age cutoffs, demographic qualifiers, or clinical subgroups. Labeling language was reviewed manually using close reading and thematic coding; no automated or NLP-based techniques were employed. Given regulatory distinctions between FDA centers, age-based restrictions were interpreted in alignment with the FDA CDRH convention, which designates adults as individuals aged 22 years or older [23].

Data analysis

All data were compiled into a structured dataset for analysis. Descriptive statistics were used to summarize trends and distributions, and findings are presented in tabular and narrative form. No patient-level data were involved in this research, and as such, institutional review board approval was not required.

Data availability

All data analyzed in this study were obtained from publicly accessible sources maintained by the FDA. No proprietary, restricted, or patient-level data were used. A structured dataset compiled from these sources is available from the corresponding author upon reasonable request.

## Results

Regulatory trajectory and market entry

**Figure 1:**
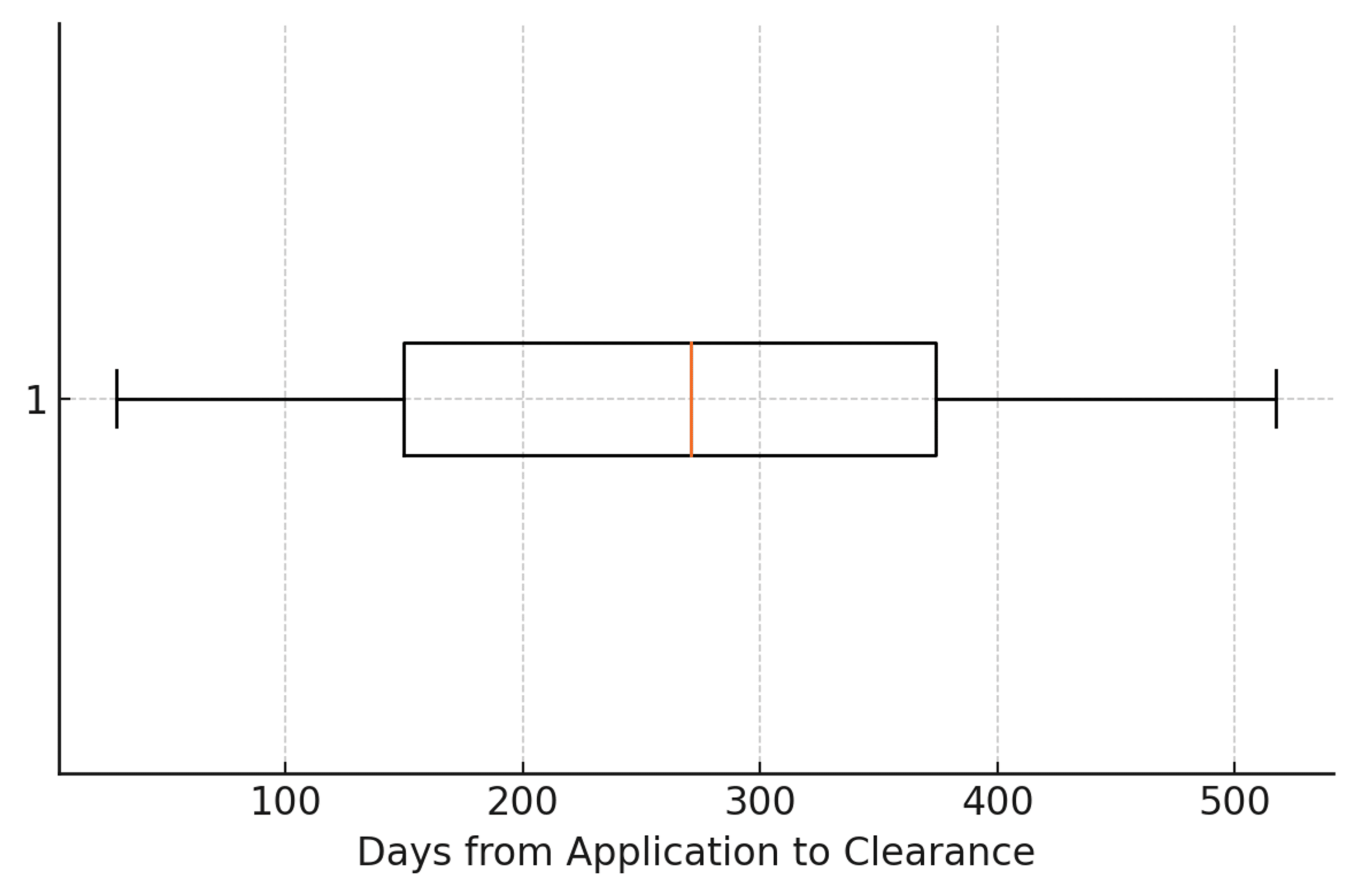
Distribution of FDA review duration for prescription digital therapeutics This author-generated boxplot illustrates the distribution of FDA review durations, measured in days from the date of application receipt to the date of regulatory clearance, for 13 prescription digital therapeutics (PDTs) cleared as of May 2025. The interquartile range represents the middle 50% of review times, while the whiskers indicate the minimum and maximum values within 1.5 times the interquartile range. The shortest review period was 29 days (Parallel), while the longest extended to 517 days (DaylightRx), reflecting variability in regulatory timelines across submissions.

A total of 13 FDA-cleared PDTs were identified as of May 2025. The majority of products (n = 8, 61.5%) were cleared through the 510(k) premarket notification pathway, while the remaining (n = 5, 38.5%) underwent de novo classification. The average time from FDA receipt of application to clearance was 266 days, with a median duration of 271 days. The shortest observed review period was 29 days for Parallel (Mahana Therapeutics Inc., San Francisco, CA, USA), while the longest spanned 517 days for DaylightRx (Big Health Inc., San Francisco, CA, USA) (Figure 1). The abbreviated review period for Parallel likely reflects its use of a closely related predicate, an earlier web-based version of the same product (Parallel, DEN200028), which enabled streamlined FDA evaluation based on substantial equivalence.

Regulatory clearances were distributed across the years 2017 to 2025, with at least one PDT cleared in 2017 (n = 1), 2018 (n = 1), 2020 (n = 2), 2021 (n = 2), 2023 (n = 2), 2024 (n = 4), and 2025 (n = 1). The year 2024 marked the peak in regulatory activity, reflecting recent momentum in the digital therapeutics space.

FDA regulation and predicate device use

The PDTs analyzed were classified under five distinct FDA regulation names after normalization for capitalization. The most common was “Computerized Behavioral Therapy Device for Psychiatric Disorders," cited in seven clearances. This regulation category encompassed DaylightRx, MamaLift Plus (Curio Digital Therapeutics, Princeton, NJ, USA), Rejoyn (Otsuka America Pharmaceutical, Inc., Rockville, MD, USA), reSET (Pear Therapeutics, Inc., San Francisco, CA, USA), reSET-O (Pear Therapeutics, Inc., San Francisco, CA, USA), SleepioRx (Big Health Inc., San Francisco, CA, USA), and Somryst (Pear Therapeutics, Inc., Boston, MA, USA), reflecting a broad application of this classification to devices targeting mood, anxiety, and substance use conditions.

The remaining four regulation names each appeared in only one clearance: “Computerized Behavioral Therapy Device for Headache” was applied to CT-132 (Click Therapeutics, Inc., New York, NY, USA), “Computerized Behavioral Therapy Device for the Treatment of Fibromyalgia” to Stanza (Swing Therapeutics, Inc., San Francisco, CA, USA), “Computerized Behavioral Therapy Device for Treating Symptoms of Gastrointestinal Conditions” to Regulora, and “Computerized Behavioral Therapy Device for Treating Symptoms” to Parallel. These labels suggest a consistent regulatory approach that adapts the core behavioral therapy device classification to more narrowly defined clinical contexts.

Nine unique FDA product codes were assigned across the 13 PDTs. Four product codes were used for more than one device. PWE was assigned to reSET and reSET-O; QMY to Parallel and Regulora; QVO to SleepioRx and Somryst; and SAP to MamaLift Plus and Rejoyn. The remaining product codes were each associated with a single device: QFT with EndeavorRx (Akili Interactive Labs Inc., Boston, MA, USA), SEE with CT-132, QXC with AspyreRx, and SCP with DaylightRx. These repeated code assignments suggest a growing consistency in the classification of digital therapeutics with overlapping therapeutic intent or technical function.

Eight of the 13 PDTs (61.5%) cited a predicate device in their FDA submissions, indicating reliance on previously cleared technologies for substantial equivalence. Two predicate devices were cited repeatedly: Somryst (K191716) served as the predicate for DaylightRx, MamaLift Plus, and SleepioRx, while reSET (DEN160018) was cited in the clearance of Rejoyn, reSET-O, and Somryst. These repeat citations underscore the foundational role of early-cleared PDTs in establishing regulatory precedent.

FDA divisions and offices

The 13 PDTs were reviewed across several FDA offices, reflecting the therapeutic diversity and multispecialty oversight of software-based treatments. The Office of Neurological and Physical Medicine Devices reviewed Rejoyn, Somryst, and Stanza, consistent with their focus on mood, sleep, and chronic pain. The Office of In Vitro Diagnostics reviewed AspyreRx, aligned with its metabolic application in type 2 diabetes, while the Office of Device Evaluation handled reSET, one of the earliest PDTs cleared.

The Office of GastroRenal, ObGyn, General Hospital, and Urology Devices was cited under two listings and reviewed Parallel and Regulora (metaMe Health, Inc., Chicago, IL, USA), both targeting gastrointestinal-related symptoms. These assignments suggest that FDA offices are selected in alignment with the intended therapeutic domain of the PDT rather than its software modality. This reflects standard FDA practice of assigning review divisions by clinical specialty rather than device type, though the cross-cutting nature of PDTs may contribute to variability in division assignment and documentation transparency.

Division-level detail was available for only nine of the 13 PDTs; the remaining four devices (30.8%) did not report the reviewing division in publicly accessible documentation. This gap highlights variability in public-facing regulatory detail and reinforces the decentralized, domain-based approach that the FDA takes in assigning digital therapeutic reviews.

Sponsor and geographic insights

The 13 cleared PDTs were developed by 10 unique sponsor companies. Most organizations had a single FDA-cleared product, while two companies accounted for multiple submissions. Pear Therapeutics, Inc. was the most prolific sponsor, with three cleared PDTs: reSET, reSET-O, and Somryst. Big Health Inc. followed with two products: DaylightRx and SleepioRx.

Sponsor headquarters were predominantly located in established U.S. hubs for biotechnology and digital health innovation. San Francisco, California, was the most represented city, with six PDTs originating from sponsors based there: DaylightRx, SleepioRx, Parallel, reSET-O, Somryst, and Stanza. Other PDT sponsors were based in Boston, MA (reSET, EndeavorRx), New York, NY (CT-132), Princeton, NJ (MamaLift Plus), Rockville, MD (Rejoyn), Monroeville, PA (AspyreRx), and Chicago, IL (Regulora).

All sponsor companies were headquartered in the US, reflecting the concentration of early digital therapeutic innovation within the domestic regulatory and investment landscape. This distribution mirrors broader patterns of venture capital investment, academic medical center affiliations, and startup formation in the digital health sector.

Indication and therapeutic area mapping

The 13 FDA-cleared PDTs targeted a broad range of clinical conditions across mental health, neurological, metabolic, and gastrointestinal domains. Most indications were non-overlapping, with each PDT focused on a distinct disease state. However, two conditions were addressed by multiple cleared products, suggesting both clinical demand and regulatory precedent in those areas.

Chronic insomnia was the most frequently targeted condition, with two PDTs - Somryst and SleepioRx - cleared to treat it. Similarly, irritable bowel syndrome was the focus of both Parallel and Regulora, representing gastrointestinal applications of digital therapeutics. All other conditions were addressed by a single PDT.

The remaining indications spanned a wide clinical spectrum: Rejoyn for major depressive disorder, MamaLift Plus for post-partum depression, reSET for substance use disorder, reSET-O for opioid use disorder, and DaylightRx for generalized anxiety disorder. Neurological and pain-related conditions were also represented, including Stanza for fibromyalgia, CT-132 for episodic migraine, and EndeavorRx for attention deficit hyperactivity disorder. AspyreRx extended PDT applications to the metabolic domain, receiving clearance for the management of type 2 diabetes.

This distribution highlights both the versatility of PDTs across organ systems and the FDA’s willingness to grant market access for diverse therapeutic indications, including traditionally underserved or comorbid conditions.

ICD-11 condition classification

Each PDT in the dataset was mapped to a corresponding International Classification of Diseases, 11th Revision (ICD-11) code to enable standardized clinical categorization. The resulting classifications spanned multiple ICD-11 chapters, underscoring the broad applicability of PDTs across medical domains. While most mappings were direct, some PDT labels used symptom-based rather than diagnostic terminology, requiring interpretive judgment to assign the most appropriate ICD-11 code.

Two ICD-11 codes were associated with more than one cleared PDT. The code 7A00 (insomnia disorder) was assigned to both Somryst and SleepioRx, reflecting the prominence of sleep disorders as early targets for digital therapeutic intervention. Similarly, DD91 (irritable bowel syndrome or certain specified functional bowel disorders) was the code assigned to both Parallel and Regulora, which focus on gastrointestinal symptom management.

The remaining PDTs each mapped to unique ICD-11 codes. Rejoyn was classified under 6A70 / 6A71 (depressive episode and Recurrent depressive disorder), while MamaLift Plus was mapped to 6A70 and MG51.1, indicating a post-partum depressive episode. reSET and reSET-O were associated with 6C40 (disorders due to substance use or addiction) and 6C43 (disorders due to use of opioids), respectively. DaylightRx was assigned to 6B00 (generalised anxiety disorder) and Stanza to MG30.01 (chronic widespread pain (fibromyalgia)).

Other unique codes included 8A80 (migraine for CT-132), 6A05 (attention deficit hyperactivity disorder for EndeavorRx), and 5A11 (type 2 diabetes mellitus for AspyreRx). These mappings demonstrate that PDTs have penetrated clinical categories ranging from neurological and metabolic disorders to pain, mood, and addiction-related conditions.

Regulatory labeling language comparison

Eleven of the 13 FDA-cleared PDTs were labeled as treatments for a specific disease or condition (Table 1). Only EndeavorRx and reSET-O did not include treatment language in their indications. EndeavorRx for pediatric attention-deficit/hyperactivity disorder did not include treatment terminology, instead emphasizing functional improvement on “sustained and selective attention and may not display benefits in typical behavioral symptoms, such as hyperactivity.” reSET-O was described as “intended to increase retention of patients with opioid use disorder in outpatient treatment,” explicitly framing its role as a adjunctive behavioral support tool.

**Table 1:** FDA-cleared indications for use of prescription digital therapeutics This author-created table summarizes the FDA-cleared indications for use (IFU) of each prescription digital therapeutic (PDT), as documented in FDA decision summaries as of May 2025. Each entry reflects the regulatory labeling approved including information on intended population, therapeutic claims (e.g., treatment, symptom reduction, or disease management), and whether the PDT is adjunctive to other clinical care. These statements form the basis of the therapeutic scope and regulatory positioning for each PDT.

PDT	Indication for use
CT-132	CT-132 is indicated for the preventive treatment of episodic migraine in patients 18 years of age and older. It is intended for adjunctive use alongside acute and/or other preventive treatments for migraine.
AspyreRx	BT-001 is a prescription-only digital therapeutic device intended to provide cognitive behavioral therapy to patients 18 years or older with type 2 diabetes. The device targets behavior to aid in the management of type 2 diabetes in patients who are under the care of a healthcare provider. BT-001 provides cognitive behavioral therapy as a treatment that should be used adjunctively with standard of care.
DaylightRx	Daylight is a prescription device delivering Cognitive Behavioral Therapy and can be made available on the order of a licensed healthcare provider. Daylight is a digital therapeutic intended to treat generalized anxiety disorder (GAD) by improving a patient's GAD symptoms as an adjunct to usual care in patients aged 22 years and older.
EndeavorRx	EndeavorRx is a digital therapeutic indicated to improve attention function as measured by computer-based testing in children ages 8-12 years old with primarily inattentive or combined-type ADHD, who have a demonstrated attention issue. Patients who engage with EndeavorRx demonstrate improvements in a digitally assessed measure Test of Variables of Attention (TOVA) of sustained and selective attention and may not display benefits in typical behavioral symptoms, such as hyperactivity. EndeavorRx should be considered for use as part of a therapeutic program that may include: clinician-directed therapy, medication, and/or educational programs, which further address symptoms of the disorder.
MamaLift Plus	MamaLift Plus is a prescription-only digital therapeutic intended to provide neurobehavioral interventions to patients 22 years of age and older, as an adjunct to clinician-managed outpatient care. MamaLift Plus treats mild to moderate postpartum depression by improving a patient's symptoms of depression.
Parallel	Parallel is a prescription-only digital therapeutic device intended to provide cognitive behavioral therapy for adults aged 22 years of age and older who have been diagnosed with Irritable Bowel Syndrome (IBS). Parallel is indicated as a 3 month treatment for patients with IBS. Parallel treats IBS by reducing the severity of symptoms and is intended to be used together with other IBS treatments.
Regulora	Regulora is a prescription-only digital therapeutic device intended to provide behavioral therapy through gut-directed-hypnotherapy for adults 22 years of age and older who have been diagnosed with Irritable Bowel Syndrome (IBS). Regulora is indicated as a 3-month treatment for patients with abdominal pain due to IBS and is intended to be used together with other IBS treatments
Rejoyn	Rejoyn is a prescription digital therapeutic for the treatment of Major Depressive Disorder (MDD) symptoms as an adjunct to clinician-managed outpatient care for adult patients with MDD aged 22 years and older who are on antidepressant medication. It is intended to reduce MDD symptoms.
reSET	reSET is intended to provide cognitive behavioral therapy, as an adjunct to a contingency management system, for patients 18 years of age and older who are currently enrolled in outpatient treatment under the supervision of a clinician. reSET is indicated as a 12 week (90 days) prescription-only treatment for patients with substance use disorder (SUD), who are not currently on opioid replacement therapy, who do not abuse alcohol solely, or who do not abuse opioids as their primary substance of abuse. It is intended to: increase abstinence from a patient’s substances of abuse during treatment, and increase retention in the outpatient treatment program.
reSET-O	reSET-O is intended to increase retention of patients with opioid use disorder (OUD) in outpatient treatment by providing cognitive behavioral therapy, as an adjunct to outpatient treatment that includes transmucosal buprenorphine and contingency management, for patients 18 years or older who are currently under the supervision of a clinician. reSET-O is indicated as a prescription-only digital therapeutic.
SleepioRx	Sleepio is a digital therapeutic intended for the treatment of chronic insomnia / insomnia disorder as an adjunct to usual care in patients aged 18 and older. Sleepio is a prescription device delivering Cognitive Behavioral Therapy for Insomnia (CBT-I) and can be made available on the order of a licensed healthcare provider.
Somryst	Somryst is a prescription-only digital therapeutic intended to provide a neurobehavioral intervention (Cognitive Behavioral Therapy for Insomnia - CBT-I) in patients 22 years of age and older with chronic insomnia. Somryst treats chronic insomnia by improving a patient’s insomnia symptoms.
Stanza	Stanza is a prescription digital therapeutic that provides Acceptance and Commitment Therapy, a form of Cognitive Behavioral Therapy, and is indicated for the treatment of fibromyalgia symptoms in adult patients.

The remaining 11 PDTs used phrases such as “intended to treat” or “treatment of” in their labeling, signaling primary therapeutic intent. Among these, AspyreRx was uniquely positioned as “to aid in the management” of type 2 diabetes, suggesting a supportive rather than curative therapeutic function. Several PDTs (DaylightRx, MamaLift Plus, Parallel, Rejoyn, Somryst, Stanza, and Regulora) focused on symptom relief, rather than disease modification, as the primary mechanism of benefit. These formulations suggest a regulatory preference for precise or narrowly scoped claims in complex or subjective clinical areas.

Only one product, CT-132, received what could be considered a clean treatment label, referring unambiguously to the treatment of episodic migraine management or symptom-focused modifiers. This more traditional structure mirrors conventional pharmaceutical labeling and may signal a higher confidence in the PDT’s therapeutic efficacy.

Age-based labeling also varied. CT-132, reSET, and reSET-O were approved for use starting at age 18. However, under FDA CDRH classification, individuals aged 18 to 21 are still considered pediatric, a category often termed late adolescents in device regulation [23]. EndeavorRx remained the only PDT explicitly cleared for pediatric use in children aged eight to 12 years.

## Discussion

This study provides the first comprehensive regulatory landscape analysis of FDA-cleared PDTs, examining product-level characteristics, sponsor patterns, clinical indications, and labeling language across the current market. Findings reveal that although PDTs are unified by their software-only, prescription-based nature, their regulatory pathways, clinical targets, and labeling strategies reflect significant heterogeneity and evolving norms.

Our analysis reveals patterns in regulatory pathways and labeling language that are consistent with recent reviews [24]. Most PDTs have entered the market through the 510(k) pathway, leveraging predicate devices like reSET and Somryst, which have become foundational within the digital therapeutic category. While this approach facilitates efficient market entry, it may inadvertently constrain innovation by incentivizing sponsors to conform to existing device descriptions and labeling language, rather than pursue novel indications or mechanisms via the De Novo pathway. A smaller subset, indeed, underwent De Novo classification, often when introducing new mechanisms or targeting underrepresented conditions. Despite this divergence, the majority of PDTs were regulated through a limited number of reviewing FDA offices, especially those overseeing neurological and behavioral health domains.

Notably, sponsor activity was heavily clustered in established US innovation hubs, particularly San Francisco, CA, which accounted for nearly half of all PDTs. This geographic concentration mirrors the broader digital health startup ecosystem and suggests that access to venture capital, technical talent, and regulatory consulting may play a role in PDT market entry [25].

Therapeutic indications covered a broad spectrum of disorders, including migraine, depression, type 2 diabetes, insomnia, substance use, ADHD, and irritable bowel syndrome. Most conditions were uniquely represented by a single PDT, although chronic insomnia and irritable bowel syndrome had multiple FDA-cleared options, highlighting early areas of clinical traction and commercial investment. These conditions often lack effective pharmacologic options yet have a robust evidence base for face-to-face behavioral interventions like CBT and acceptance and commitment therapy (ACT). Because access to such therapies remains limited in real-world settings, their digital translation through PDTs may explain greater regulatory receptivity. This clustering likely reflects a convergence of strong market demand, persistent unmet clinical need, and FDA familiarity with cognitive and behavioral interventions for these conditions.

Labeling language analysis revealed strategic variation in how PDTs are positioned within their respective indications. While all but one PDT were described as treating a disease or condition, several had labels that emphasized symptom reduction or disease management, rather than definitive treatment claims. These distinctions illustrate how PDT sponsors navigate FDA frameworks to balance therapeutic intent with regulatory expectations. This cautious wording may reflect either regulatory conservatism around software-based claims or actual limitations in clinical trial endpoints, particularly in subjective or multifactorial conditions where hard clinical outcomes are difficult to measure. The choice of phrasing (treatment, management, or symptom relief) can influence prescriber adoption, payer interpretation, and patient expectations, particularly in indications where clinical outcomes are subjective or multifactorial [3,26]. Furthermore, age-based population labeling varied in ways that may reflect intended use cases, study populations, or regulatory conservatism. Only CT-132 for episodic migraine received a traditional treatment label for a disease without management or symptom-modifying qualifiers. EndeavorRx remains the only PDT explicitly cleared for pediatric use. Its indication is narrowly framed around attention function in children and relies on digital performance metrics, suggesting it is more of a regulatory exception than a precedent for broader pediatric digital therapeutic approval.

Taken together, these findings illustrate the emergence of a novel class of therapeutics defined not only by software and prescription status but also by their regulatory complexity, strategic positioning, and cross-domain applicability.

Labeling exceptions and regulatory flexibility

Two PDTs, reSET-O and EndeavorRx, stand out for their departure from conventional disease treatment labeling. reSET-O is not indicated to treat opioid use disorder, but rather “to increase retention of patients in outpatient treatment,” positioning it as a behavioral adherence tool adjunctive to buprenorphine and contingency management. EndeavorRx similarly avoids a direct disease treatment claim, instead being indicated “to improve attention function” in children with ADHD, as measured by computerized testing. Notably, the label cautions that benefits may not extend to core behavioral symptoms such as hyperactivity.

These atypical indications reflect the early stage of regulatory paradigm development for PDTs at the time these products were cleared [27]. Both were authorized during a period when FDA frameworks for digital therapeutics were still emerging, and the agency’s tolerance for functional or behavioral endpoints may have reflected a flexible, exploratory stance.

More recent clearances, such as CT-132 for migraine, exhibit a sharper regulatory formulation, including a clear disease treatment claim and defined population labeling. This evolution suggests increasing confidence in PDT efficacy, a maturing regulatory understanding of software-only interventions, and a shift toward greater alignment with drug-like therapeutic models.

These outliers not only demonstrate the FDA’s adaptive approach to novel digital tools but also chart the trajectory from early accommodation of behavioral support technologies to more formalized therapeutic claims grounded in evidence and precedent.

Implications for PDT development and regulation

The findings of this study underscore the importance of strategic regulatory navigation in the development and positioning of PDTs. The frequent reuse of predicate devices, particularly reSET and Somryst, has created a pathway of least resistance for subsequent 510(k) clearances. However, this reliance on predicate lineage may limit innovation if developers are incentivized to remain within existing regulatory templates rather than pursue novel mechanisms via the De Novo pathway. While alignment with established device codes and labeling conventions can streamline coverage decisions and facilitate provider adoption, it may also constrain clinical differentiation and discourage risk-taking in design or trial endpoints.

Labeling language plays a critical role not only in FDA clearance but also in downstream clinical and commercial adoption. Terms such as “treat,” “manage,” “reduce symptoms,” or “aid in” carry implications for how payers assess value, how clinicians interpret efficacy, and how patients perceive legitimacy [28-30]. The fact that most PDTs avoid the unqualified “treatment of disease” language, except for CT-132, may reflect a regulatory tendency to align claims with trial endpoints and clinical evidence thresholds. However, this ambiguity can create challenges for reimbursement and clinical guideline inclusion, particularly when PDTs are intended to adjuncts or even supplant pharmaceutical treatments.

The geographic clustering of PDT sponsors in digital health hubs also has policy implications. While concentrated innovation can drive standards and accelerate iteration, it may reinforce disparities in access to capital, regulatory expertise, and FDA engagement. Encouraging distributed development models, through regional accelerators, public-private partnerships, or telehealth-PDT delivery, may help expand participation in the PDT ecosystem. Implementation strategies could include NIH SBIR/STTR grants targeting digital therapeutics, rural innovation programs that foster technology development outside major hubs, and enhanced FDA guidance or mentorship for first-time device applicants.

Limitations

This study is subject to several limitations. First, the analysis was based entirely on publicly available FDA documentation, including de novo decision summaries, 510(k) clearance letters, and product classification listings. These records may omit or inconsistently report key regulatory metadata, such as reviewing divisions, application version histories, or trial design specifics, and do not uniformly include labeling changes post-clearance. To address this, the FDA could consider standardizing sponsor metadata and reviewing office attribution across public regulatory documents to support transparency and research reproducibility. Second, this research focused exclusively on FDA-cleared PDTs regulated by the CDRH. Software-based therapeutics that were approved via alternative regulatory centers (e.g., Center for Drug Evaluation and Research and Center for Biologics Evaluation and Research), combined with hardware components (e.g., virtual reality headsets), or authorized under emergency use or investigational exemptions were excluded. As a result, the findings may not generalize to the full spectrum of software-enabled therapies or global digital therapeutic markets.

Third, this study did not evaluate the clinical trial quality, strength of evidence, or real-world performance of the included products. While product labeling was systematically reviewed for therapeutic intent and symptom focus, no formal assessment of efficacy or outcome data was conducted. This limitation was intentional to preserve focus on regulatory characterization, but it introduces the risk that labeling language may not fully reflect clinical differentiation or impact. Finally, while efforts were made to standardize classification using tools such as ICD-11 and harmonized terminology, categorization of indications and sponsor attributes may still reflect minor subjective interpretation due to variability in labeling specificity and company naming conventions across FDA filings.

Future directions

As the PDT field matures, further research should examine correlations between labeling language and clinical outcomes, including whether phrasing influences adoption, adherence, or payer decisions. Mixed-methods studies that integrate claims data, clinician interviews, and patient-reported outcomes may offer a more comprehensive understanding of how labeling language affects real-world uptake and perceived value. Ongoing tracking of FDA clearances, predicate reuse patterns, and updates to regulation names or product codes may also offer insights into the formalization of digital therapeutic categories within the US regulatory infrastructure. Finally, the development of a standardized labeling framework for PDTs, potentially akin to drug class monographs, could help reduce ambiguity and support more consistent interpretation by clinicians, payers, and patients alike. Such efforts could be led by the FDA in collaboration with professional medical societies and/or health IT standards organizations such as HL7, which have experience developing and maintaining interoperable clinical terminologies.

## Conclusions

This study provides the first comprehensive analysis of FDA-cleared PDTs, revealing key trends in regulatory strategy, therapeutic positioning, and sponsor landscape. While all PDTs share a software-only and prescription-based foundation, their labeling language, intended use claims, and regulatory pathways reflect substantial diversity. The distinction between treatment, symptom relief, and management claims, alongside age-based population differences, illustrates a maturing regulatory paradigm that has evolved from early functional and behavioral tools to more direct therapeutic claims. These findings, based on publicly available FDA documentation as of May 2025, underscore the importance of regulatory language as both a scientific and strategic asset in digital medicine and offer a blueprint for future PDT developers, specifically in label construction, predicate use strategy, and target indication selection.
